# Amyloid-β Impairs Dendritic Trafficking of Golgi-Like Organelles in the Early Phase Preceding Neurite Atrophy: Rescue by Mirtazapine

**DOI:** 10.3389/fnmol.2021.661728

**Published:** 2021-06-03

**Authors:** Elsa Fabbretti, Giulia Antognolli, Enrico Tongiorgi

**Affiliations:** Department of Life Sciences, University of Trieste, Trieste, Italy

**Keywords:** neurodegenerative disorders, amyloid-beta, neurite atrophy, neuronal polarity, Golgi-like organelles, intracellular vesicle trafficking, antidepressants, dendritic arborization

## Abstract

Neurite atrophy with loss of neuronal polarity is a pathological hallmark of Alzheimer’s disease (AD) and other neurological disorders. While there is substantial agreement that disruption of intracellular vesicle trafficking is associated with axonal pathology in AD, comparatively less is known regarding its role in dendritic atrophy. This is a significant gap of knowledge because, unlike axons, dendrites are endowed with the complete endomembrane system comprising endoplasmic reticulum (ER), ER–Golgi intermediate compartment (ERGIC), Golgi apparatus, post-Golgi vesicles, and a recycling-degradative route. In this study, using live-imaging of pGOLT-expressing vesicles, indicative of Golgi outposts and satellites, we investigate how amyloid-β (Aβ) oligomers affect the trafficking of Golgi-like organelles in the different dendritic compartments of cultured rat hippocampal neurons. We found that short-term (4 h) treatment with Aβ led to a decrease in anterograde trafficking of Golgi vesicles in dendrites of both resting and stimulated (with 50 mM KCl) neurons. We also characterized the ability of mirtazapine, a noradrenergic and specific serotonergic tetracyclic antidepressant (NaSSA), to rescue Golgi dynamics in dendrites. Mirtazapine treatment (10 μM) increased the number and both anterograde and retrograde motility, reducing the percentage of static Golgi vesicles. Finally, mirtazapine reverted the neurite atrophy induced by 24 h treatment with Aβ oligomers, suggesting that this drug is able to counteract the effects of Aβ by improving the dendritic trafficking of Golgi-related vesicles.

## Introduction

Neurite atrophy, including abnormal morphology and retraction or loss of part of the dendritic and/or axonal arborization, is a pathological symptom underlying several neurodevelopmental and neurodegenerative brain diseases such as autism, Rett syndrome (RTT), Fragile X syndrome, schizophrenia, and Alzheimer’s disease (AD) (Kaufmann and Moser, 2000; Kulkarni and Firestein, 2012). Among the possible causes of neurite atrophy is the impairment of microtubules-based motor machineries involved in vesicle and protein anterograde trafficking toward distal neurite endings, as well as in retrograde vesicle recycling mechanisms (Overk and Masliah, 2014; Dubey et al., 2015; De Vos and Hafezparast, 2017). Trafficking mechanisms are necessary for the maintenance of the integrity and spatial organization of the secretory pathway which, in turn, is essential for stability of axons and dendrites, and synaptic function (Ramírez and Couve, 2011; Koleske, 2013; Maeder et al., 2014). Indeed, several proteins involved in the regulation of either secretory membrane trafficking or the endocytic pathway were identified by GWAS (genome wide sequencing studies) as susceptibility genes in AD (Toh and Gleeson, 2016). While there is substantial agreement that anterograde axonal transport of synaptic secretory vesicles and both anterograde and retrograde axonal traffic of endocytic vesicles are impaired in AD, comparatively less is known about how AD affects the dynamics of secretory pathway organelles in dendrites.

Unlike axons, dendrites are endowed with the complete endomembrane system comprising the forward biosynthetic route constituted by the endoplasmic reticulum (ER), the ER–Golgi intermediate compartment (ERGIC), the Golgi apparatus and post-Golgi vesicles, and a recycling-degradative route constituted by endosomes and lysosomes (Pierce et al., 2001; Ramírez and Couve, 2011). All membranous elements of the secretory pathway show dynamic rearrangements that are critical for dendrites outgrowth and neuronal polarization during development, as well as synaptic plasticity and homeostasis in adulthood (Horton and Ehlers, 2004; Tang, 2008). However, the different secretory organelles display different localization and degrees of motility. The ER forms an anatomizing network distributed throughout the entire dendritic arbor with local zones of increased complexity at dendritic branching points and upper order dendrites, in which highly dynamic domains are involved in local ER-to-Golgi protein export (Aridor et al., 2004; Cui-Wang et al., 2012). In contrast, stacks of Golgi cisternae with no connection to the somatic Golgi, also designed as Golgi outposts (GO), are stably localized in the first two segments of large dendrites (primary and secondary dendrites) and at branching points between primary/secondary and secondary/tertiary dendrites (Horton and Ehlers, 2003a; Tongiorgi, 2008). More dynamic *Trans-*Golgi network (TGN) compartments were discovered in distal dendrites where they undergo rapid anterograde and retrograde movements (Horton and Ehlers, 2003a; McNamara et al., 2004; Tongiorgi, 2008). In distal dendrites, the high dynamic features of the trafficking of these post-Golgi organelles are similar to the pre-Golgi ERGIC vesicles and to other more recently discovered Golgi-like small cisternae, known as Golgi satellites that can be identified by the expression of the pGOLT protein (Mikhaylova et al., 2016).

Aberrant folding and accumulation of the amyloid-β peptide (Aβ), an hallmark of AD, causes neurite degeneration, synapse loss, and impairment in neuronal trafficking (Serrano-Pozo et al., 2011; Plá et al., 2017). In the present study, using a live imaging approach on hippocampal neurons in culture, we investigate how Aβ_25__–__25_ oligomers affect the dynamics of pGOLT-expressing vesicles indicative of Golgi-related organelles, such as Golgi outposts and satellites (Horton and Ehlers, 2003b; Horton et al., 2005; Mikhaylova et al., 2016). We also characterize the ability of mirtazapine, a noradrenergic and specific serotonergic tetracyclic antidepressant (NaSSA), to rescue Golgi trafficking. The neurotrophic effect of antidepressants is well known and demonstrated in several pathological models (Castrén, 2004), and we have previously shown that mirtazapine can rescue dendritic atrophy and soma size shrinkage of cortical and hippocampal neurons in a mouse model of Rett syndrome (Bittolo et al., 2016; Nerli et al., 2020).

## Materials and Methods

### Primary Cultures of Rat Hippocampal Neurons

Animals were treated according to the institutional guidelines, in compliance with the European Community Council Directive 2010/63/UE for care and use of experimental animals. Authorization for animal experimentation was obtained from the local ethical committee on November 10, 2017 and was communicated to the Italian Ministry of Health, in compliance with the Italian law D. Lgs. 116/92 and the L. 96/2013, art. 13. All efforts were made to minimize animal suffering and to reduce the number of animals used. Hippocampal neurons were prepared from postnatal day 0 to 1 (P0–P1) Wistar rats as previously described (Baj et al., 2014). Cultures were maintained in Neurobasal medium (Life Technologies) supplemented with B27 (Thermo Fisher Scientific, Waltham, MA, United States), 1 mM L-glutamine and antibiotics (Euroclone, Milan, Italy) on 24-well imaging plates (Eppendorf, Hamburg, Germany) or glass coverslips pre-coated with poly-L-ornithine (100 μg/ml) and Matrigel^TM^ (Corning, NY, United States). At days *in vitro* 3 (DIV3), cytosine 2.5 μM β-D-arabinofuranoside was added. For transfection experiments, neurons were used at a concentration of 200 cells/mm^2^, used at DIV6 and analyzed 24 h later. For neurite outgrowth analysis, neurons were seeded at a concentration of 320 cell/mm^2^.

### Ab_25__–__35_ and Mirtazapine Treatment

Aggregation of Aβ_25__–__35_ peptide (5 mg/ml, Bachem, Bubendorf, Switzerland; Copani et al., 1995) was obtained in phosphate buffer for 24 h at 37°C (Millucci et al., 2010). A-beta25–35 oligomers aggregation was previously demonstrated by atomic force microscopy, showing formation of oligomeric/protofibrillary assemblies displaying the typical beta-sheet structure (Antonini et al., 2011). Aggregated Aβ oligomers (10 μM; Gomes et al., 2014) were applied on DIV7 hippocampal cultures for 4–24 h. Mirtazapine (10 μM in DMSO; Abcam, Cambridge, United Kingdom; Fukuyama et al., 2013; Bittolo et al., 2016) was applied alone or co-applied with Aβ on DIV7 cultures. Control neurons were treated with vehicle only (DMSO). Effective concentrations of Aβ and mirtazapine (24 h) were initially chosen accordingly to data from a (3-(4,5-dimethylthiazol-2-yl)-2,5-diphenyltetrazolium bromide) tetrazolium (MTT) reduction assay performed on DIV7 hippocampal cultures. Short (4 h) incubation of hippocampal neurons with Aβ peptide (10 μM) was sufficient to significantly impair mitochondrial membrane potential with respect to control (Supplementary Figure 1).

### Immunofluorescence and Images Acquisition

For immunofluorescence experiments, hippocampal cultures were fixed in 4% paraformaldehyde for 15 min at room temperature and incubated for 1 h with the following primary antibodies diluted in phosphate buffer with 0.1% Triton-X100 and 2% of bovine serum albumin: anti-β Tubulin III (1:1000; Sigma, Milan, Italy), anti-NeuN (1:1000; Millipore, Burlington, MA, United States), anti-GM130 (1:250; BD Transduction Laboratories, San Jose, CA, United States), anti-TGN38/46 (1:250; Abcam, Cambridge, United Kingdom), and anti-LMAN1 (1:250; Sigma, Milan, Italy). Immunolabeling was visualized with 488-/568-AlexaFluor-conjugated secondary antibodies (1:500; Thermo Fisher Scientific). Nuclei were counterstained with Hoechst 33342 (0.001%; Thermo Fisher Scientific) and visualized with a Nikon ECLIPSE T*i*-E epifluorescence microscope or an ECLIPSE C1si confocal microscope (Nikon, Tokyo, Japan). Fields were captured at a resolution of 1 pixel = 0.44 micron, using the Nikon acquisition software NIS-elements, a 40x DIC H 0.17, and an oil immersion objective with a working distance of 160 μm. NeuN- and Hoechst-positive cells were counted using the Object Analyzer option of the NIS-Elements.

### Total Neurite Length (TNL) Analysis

An automated analysis approach was used to quantify the neurite retraction and recovery after 24 h of treatment. This automated analysis was performed using the open source bioinformatics toolkit NeuriteQuant, implemented in the free image analysis software ImageJ/Fiji (Dehmelt et al., 2011; Nerli et al., 2020). Following a staining of neurites using an antibody against cytoskeletal proteins such as Tubulin-β-III or MAP-2, this tool allows for the measurement of total neuritic or dendritic length on primary neuronal cultures. Moreover, by analyzing multi-channel images, it allows researchers to measure many other parameters such as total neuronal cell body area, total number of cell bodies, number of neurite-cell body attachment points, and number of neurite endpoints. To perform the analysis, 8-bit images are necessary. The NeuriteQuant analysis settings were set by a configuration file, in which the parameters for the analysis were as follows: Neurite detection width = 12; neurite detection threshold = 10; neurite cleanup threshold = 150; neurite cell body detection = 80.

### Transfection of Hippocampal Neurons and Live Imaging Experiments

**FIGURE 1:**
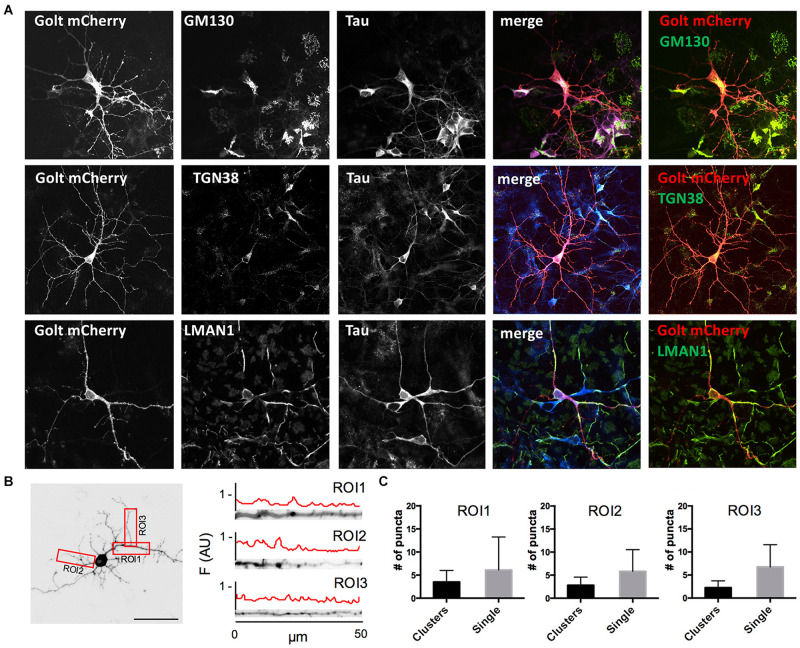
Characterization of pGOLT signals in hippocampal neurons cultured for 7 days *in vitro*. **(A)** Representative confocal images of pGOLT-mCherry transfected neurons (red), Tau cytoskeletal marker (blue) combined with immunofluorescence (green) for either Golgi marker GM130 **(top)**, *trans-*Golgi network marker TGN38 **(mid)**, or endoplasmic reticulum marker LMAN1 **(bottom)**. Transfected neurons present a diffuse and spotted pGOLT-mCherry expression, with high intensity staining in the somatic region. Scale bars = 5 μm. **(B)** **Left**. Image converted in gray levels and B/W inverted of one neuron expressing low pGOLT levels. Each red rectangle represents a 50 μm-long region of interest (ROI). ROI1 corresponds to the proximal part of the apical dendrite; ROI2 to the proximal part of basal dendrite; and ROI3 to a higher order process. **(B)** **Right**. Straightened dendrites presenting several pGOLT spots of variable dimensions. Fluorescence is analyzed as arbitrary units in each segment by an intensity profile plot [F(AU), red line]. Peaks of the intensity plot profile coincide with pGOLT-related vesicles. **(C)** Average number of persistent pGOLT clusters or single puncta per segment in unstimulated neurons. RO1 = proximal apical, RO2 = proximal basal, RO3 = distal apical. *N* = 10 neurons per each condition.

Plasmid transfection was done on DIV6 hippocampal neurons with Lipofectamine 2000^TM^ (Thermo Fisher Scientific) with pEGFP (Takara, Clontech) or pGOLT-mCherry (Addgene plasmid # 73297; Mikhaylova et al., 2016) and transfected cells were used 16 h later. For live imaging experiments, only low expressing pGOLT neurons with punctate fluorescence were selected while pGOLT high expressing neurons were excluded. pGOLT-positive neurons were selected from low magnification large-fields and analyzed individually with a 40x objective in a solution containing 5.3 mM KCl, 50.9 mM NaCl, HEPES 10.9 mM, NaHPO_4_ 0.8 mM, NaHCO_3_ 26 mM, MgCl_2_ 0.8 mM, CaCl_2_ 1.8 mM, and glucose 25 mM, under temperature and CO_2_ atmosphere control (Nikon). Sequential time lapse images were acquired in 1 min-time intervals for a total of 10 min, with a CMOS Nikon DS-Qi2 camera at a 500–700 s exposure time and with neutral density filter 4, in order to minimize the phototoxicity. For tests at high K^+^, cultures were stimulated with an isotonic solution containing 50 mM KCl and immediately analyzed for time lapse recording for 5 or 10 min. For kymographs, neurons were imaged in 5 s-time intervals for 2 min. Fluorescence peak analysis of pGOLT spots was obtained with ImageJ/Fiji, along 50 μm-long line selections (ROIs) from proximal apical, proximal basal dendrites, and higher order dendrites (Figure 1B). The peak values from each time were analyzed in Microsoft Office Excel. Peaks of similar intensities found in an adjacent space and persisting for more than 3 min were defined as “persistent spots.” Peaks oscillating in a ±0.22 μm space were defined as “static.” Only long-lasting persistent pGOLT spots were used for further analysis. A mobility index was defined as the net distance traveled by individual persistent pGOLT spots (μm) obtained by summing all anterograde and retrograde movements covered by pGOLT spots between the first and last step of a 10 min observation time.

### Statistical Analysis

Statistical analysis was performed using Prism 5 software (GraphPad). Based on D’Agostino and Pearson’s omnibus positive normality test, statistical significance between groups was obtained with student’s *t*-test, or with the Mann–Whitney Rank Sum test. For multiple comparisons, one-way ANOVA or Kruskal–Wallis statistical analysis were performed. Data are represented as average percentage ± standard error mean (SEM).

## Results

### Dendritic Trafficking of Golgi-Like Organelles

Golgi organelles are present in the soma and dendrites, but not in axons, and can be labeled in living neurons by overexpressing the recombinant protein pGOLT-mCherry (pGOLT) (Mikhaylova et al., 2016). To confirm the effective identification of Golgi organelles by pGOLT, we verified by confocal analysis in pGOLT-mCherry-transfected primary rat hippocampal neuronal cultures at 7 days *in vitro* (DIV7), if pGOLT-mCherry-labeled organelles were also immunostained by antibodies specific for the Golgi outposts protein GM130, or the *trans-*Golgi network marker TGN38/46, or the ERGIC-specific mannose lectin LMAN1/ERGIC-53. We used 7-day *in vitro* neurons because young neurons (DIV 6–9) are more indicated than the more mature ones (DIV 15–20) for studies on Golgi trafficking since Golgi-like organelles are more abundant at younger ages while dynamics are comparable at all ages (Mikhaylova et al., 2016).

We found that pGOLT expression was mainly located in somatic and dendritic vesicles that extensively co-localized with the Golgi-cisternae marker GM130 and the *trans-*Golgi network marker TGN38/46, and to a lesser extent with the ERGIC marker LMAN1 (Figure 1A). To study the subcellular distribution of pGOLT-positive vesicles, living neurons expressing low pGOLT levels were analyzed in the three distinct 50 μm-long regions of interest (ROI) corresponding to proximal basal (ROI1), proximal apical (ROI2), and distal apical higher order processes (ROI3) (Figure 1B). We observed different types of pGOLT-labeled vesicles that we classified as follows: “Transient single puncta” with short-lived spot appearance (<3 min); “long-lasting puncta” = mobile spots covering a space >2 microns during the observation time of 10 min, and persisting for about 8–10 min; “clusters” = peaks of intensity similar to single puncta present in a contiguous space, and visible continuously for a minimum of 3 min; and “static pGOLT puncta or clusters” = stable peaks, i.e., oscillating within a space of ±0.22 microns and stable for more than 3 min. Only pGOLT-labeled long-lasting single puncta (just named single puncta) and clusters that were visualized as individual spots (pGOLT spots) and persisted for at least 3 min were further analyzed. In basal conditions, neurons exhibited large pGOLT spots (average diameter 2 ± 0.56 μm) and their average number was comparable in the three different ROIs (Figure 1C).

### Short-Term Aβ Treatment Impairs Trafficking of pGOLT-Positive Golgi Vesicles

**FIGURE 2:**
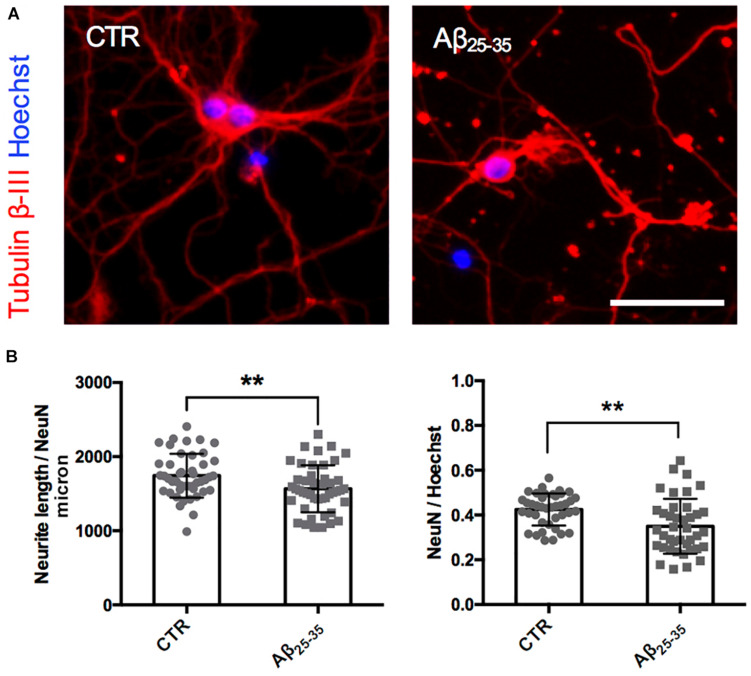
Neurite atrophy in an *in vitro* model of Aβ injury. **(A)** Representative microscopy images (10X) of DIV7 hippocampal neurons in control condition **(left)** and after 24 h of Aβ_25__–__35_ (10 μM) treatment **(right)** stained with anti-Tubulin β-III (red) and Hoechst (blue). Scale bar = 50 μm. **(B)** Scatter plot of total neurite length (TNL) for each neuron **(left)** expressed in μm/neuron, and neuronal density expressed as the ratio between the number of neurons on the total number of cells **(right)**. Each dot represents TNL from an image (10X) in control and after 24 h of Aβ_25__–__35_ (10 μM) treatment, *n* = 40–45 fields per condition from three independent experiments. Following D’Agostino and Pearson’s normality test, we performed an unpaired *t*-test. **p < 0.01.

After having established the methodology to identify Golgi-like organelles in dendrites, we asked whether Aβ treatment could impair trafficking of pGOLT spots during the early phase of the Aβ-induced injury that precedes neurite atrophy and cell death. Toward this aim, we first verified the ability of Aβ_25__–__35_ oligomers to induce neurite atrophy over 24 h in DIV7 neurons. The concentration of Aβ was chosen based on data from a (3-(4,5-dimethylthiazol-2-yl)-2,5-diphenyltetrazolium bromide) tetrazolium (MTT) reduction assay performed on DIV7 hippocampal cultures (Supplementary Figure 1). Primary rat hippocampal neuronal cultures (DIV7) were incubated with aggregated Aβ_25__–__35_ peptide (10 μM) and 24 h later, neurons were labeled by immunofluorescence for the neuron-specific microtubule protein β-Tubulin III which labels both axonal and dendritic processes (Figure 2A). Aβ-induced neurite atrophy measured using the software NeuriteQuant (see section “Materials and Methods”) was applied on fluorescence microscopy images for β-Tubulin III to quantify the total neurite length (TNL) in each condition (Figure 2B left). While control neurons presented a complex and dense neurite network, Aβ-treated neurons showed an aberrant neurite morphology with a significant TNL reduction (1743 ± 44 μm/neuron in control vs. 1566 ± 47 μm/neuron after Aβ; *n* = 3, *p* = 0.008; Figure 2B left), in line with previous observations (Resende et al., 2007). Aβ also significantly reduced neuronal density (Figure 2B right) with a significant lower number of NeuN-positive neurons, indicative of neuronal cell loss (ratio NeuN/total cells: 0.42 ± 0.01 in control vs. 0.35 ± 0.02 after Aβ; *n* = 3; *p* = 0.0013).

**FIGURE 3:**
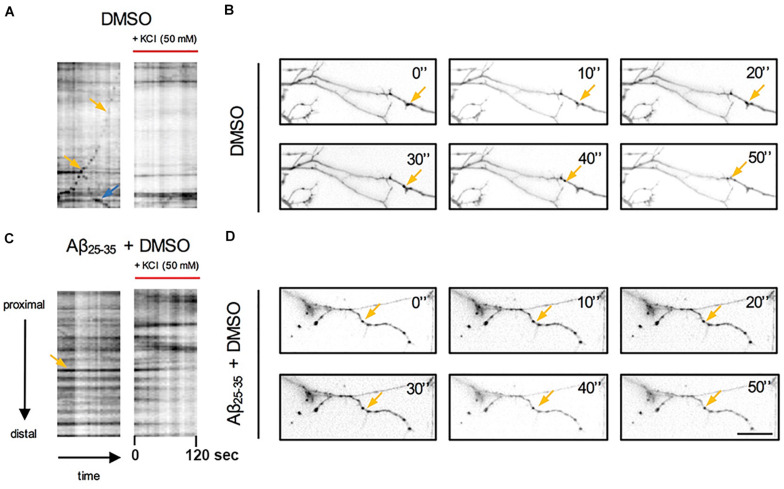
Qualitative analysis of the effects of Aβ and KCl-induced neuronal activity on pGOLT spots trafficking. Kymograph representation of **(A)** pGOLT spots trafficking (2 min) in hippocampal neurons in control (DMSO) or **(C)** Aβ treatment (10 μM, 4 h) in basal and KCl conditions (50 mM, 2 min). **(B)** Representative images of neuronal dendrites in control and **(D)** Aβ + DMSO (bottom) in basal conditions. **(A,B)** In control conditions, retrogradely (yellow arrows) and anterogradely (blue arrows) moving pGOLT spots were detected. **(C,D)** In Aβ-treated cultures, static pGOLT spots (yellow arrows) were mostly detected. Images are acquired for 2 min with 5 s interval. Scale bar = 15 μm.

**FIGURE 4:**
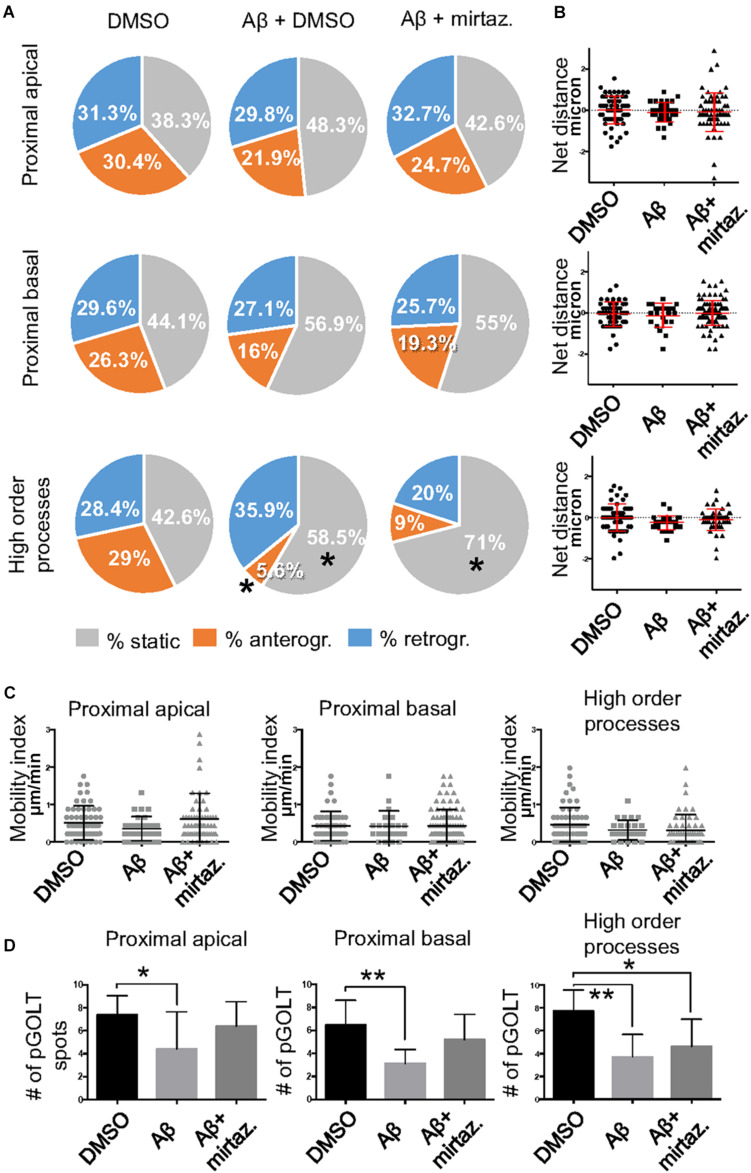
Effects of Aβ and mirtazapine on pGOLT vesicle trafficking in unstimulated cultures. **(A)** Pie charts representation of the average percentage of anterograde, retrograde, and static pGOLT spots for each cell in cultures treated with Aβ and mirtazapine (both 10 μM, 4 h). Data were obtained from 4 to 5 ROI segments in proximal apical, proximal basal, and higher order processes. DMSO: *n* = 8; Aβ + DMSO: *n* = 7; Aβ + mirtazapine: *n* = 10. **(B)** Scatter plot representation of net distance covered by pGOLT spots in the basal condition in proximal apical, proximal basal, and higher order processes after 4-h treatments. **(C)** Mobility index represents the velocity of pGOLT spots in the different segments analyzed, expressed in μm/min, after 4 h of treatments. **(D)** Average of the number of pGOLT spots in the different segments. DMSO: *n* = 8; Aβ + DMSO: *n* = 7; Aβ + mirtazapine: *n* = 10. Following D’Agostino-Pearson’s normality test, we performed an unpaired *t*-test. *p ≤ 0.05; **p ≤ 0.01.

To explore the impact of Aβ on the trafficking of Golgi vesicles, we incubated DIV7 neurons with Aβ_25__–__35_ peptide for 4 h and we investigated how it affected Golgi cargo trafficking during this early time-window that preceded neuronal atrophy and cell death. In these experiments, in order to perform further comparisons with pharmacological treatments, control cultures were treated for 4 h with DMSO which is the vehicle used to dilute mirtazapine in the subsequent set of rescue experiments. The effects of DMSO and Aβ_25__–__35_ peptide were investigated in both basal conditions and after KCl stimulation (Figure 3). Qualitative kymograph analysis of 50 μm-long proximal regions of neurons treated with DMSO showed the occurrence of both anterograde and retrograde pGOLT spots trafficking (Figures 3A–D). However, both Aβ (10 μM, 4 h) and KCl (50 mM, 2 min) treatments apparently reduced trafficking (Figures 3A,C). To better define this effect, we carried out a quantitative analysis of pGOLT spots trafficking. Neurons treated with DMSO showed a similar proportion of static, retrograde, or anterograde pGOLT spots in all the ROIs analyzed (Figure 4A and Table 1). Short Aβ treatment in DMSO (4 h) of hippocampal neurons showed a small, not significant increase of static vesicles in proximal apical dendrites and proximal basal dendrites along with a larger, statistically significant increase of +15.9% (from 42.6 ± 5.0% to 58.5 ± 13.0%, *p* < 0.05) in higher order processes (Figure 4A and Table 1). Interestingly, while the percentage of pGOLT spots undergoing retrograde trafficking was substantially unmodified, the anterograde mobility after Aβ + DMSO treatment was reduced with respect to control conditions in the different regions considered, with a small, insignificant reduction in the proximal apical (21.9 ± 8.8%) and proximal basal dendrites (16.0 ± 5.9%) and a stronger, statistically significant (*p* = 0.02) reduction of −23.4% in the higher order processes (from 29.0 ± 7.0% to 5.6 ± 3.0%) (Figure 4A and Table 1).

**TABLE 1:** Summary of the pGOLT dynamics in unstimulated cultures.

		**DMSO**	**Aβ + DMSO**	**Aβ + mirtazapine**
Proximal apical	Static	38.3 ± 5.5%	48.3 ± 9.5%	42.6 ± 8.8%
	Anterograde	30.4 ± 11.0%	21.9 ± 8.8%	24.7 ± 6.4%
	Retrograde	31.3 ± 8.4%	29.8 ± 13.0%	32.7 ± 6.0%
Proximal basal	Static	44.1 ± 9.5%	56.9 ± 13.0%	55.0 ± 5.0%
	Anterograde	26.3 ± 9.0%	16 ± 5.9.0%	19.3 ± 4.5%
	Retrograde	29.6 ± 9.7%	27.1 ± 10.0%	25.7 ± 6.0%
Higher order	Static	42.6 ± 5.0%	58.5 ± 13.0%*^1^	71 ± 6.0%*^2^
	Anterograde	29 ± 7.0%	5.6 ± 3.0%*^3^	9 ± 3.0%*^4^
	Retrograde	28.4 ± 6.0%	35.9 ± 12.0%	20 ± 5.0%

*Average percentage ± standard error of the mean (SEM) of static, anterograde, and retrograde vesicles for each neuron in control (DMSO) or after treatment with Aβ or Aβ + mirtazapine. ^∗^1: *p* < 0.05, for Aβ + DMSO vs. DMSO. ^∗^2: *p* = 0.004, for Aβ + mirtazapine vs. DMSO for static spots in high order processes. ^∗^3: *p* = 0.02, for Aβ vs. DMSO for anterograde spots in high order processes. ^∗^4: *p* = 0.02, for Aβ + mirtazapine vs. DMSO for anterograde spots in high order processes.*

Considering the space covered by individual pGOLT spots in 10-min observation times, similar net distances were measured in proximal apical, proximal basal, and higher order segments in all conditions for both anterograde and retrograde movements (Figure 4B). Of note, the net distance covered either by anterogradely or retrogradely moving pGOLT spots was not affected by the Aβ + DMSO treatment, although the population variance appeared reduced (Figure 4B). To further investigate the pGOLT spot velocity, the net distances were pooled together independently of the direction, creating a different representation of the data that we called “mobility index.” Accordingly, the mobility index (Figure 4C) and the average velocity of trafficking pGOLT spots remained substantially unchanged after Aβ + DMSO treatment, being 0.05 ± 0.007 μm/min in proximal apical, 0.05 ± 0.010 μm/min in proximal basal, and 0.08 ± 0.001 μm/min in higher order dendrites under basal conditions (Figure 4C and Table 2). Interestingly, in all segments measured, 4-h treatment with Aβ + DMSO significantly reduced the number of visible pGOLT spots with respect to control conditions (Figure 4D). In conclusion, short-term Aβ + DMSO treatment in unstimulated neuronal cultures induced a decrease of visible pGOLT spots and a reduction in the anterograde trafficking of pGOLT spots in distal high order dendrites.

**TABLE 2:** Summary of the pGOLT spot velocities (μm/min).

**Conditions**	**Proximal apical**	**Proximal basal**	**Higher order**
**DMSO**	0.05 ± 0.005	0.05 ± 0.006	0.05 ± 0.004
**A**β	0.05 ± 0.007	0.05 ± 0.010	0.08 ± 0.010
**A**β **+ Mirta**	0.09 ± 0.010*^1,2^	0.06 ± 0.007	0.08 ± 0.020

*^∗^1: *p* = 0.0088 for Aβ + Mirta vs. DMSO in proximal apical processes. ^∗^2: *p* = 0.0182 for Aβ + Mirta vs. Aβ in proximal apical processes.*

### Mirtazapine Recues Aβ-Induced Impaired Traffic and Number of pGOLT Spots in Dendrites

To explore for possible protecting effects of mirtazapine on Aβ-induced insult in hippocampal neurons, we tested whether mirtazapine (10 μM, 4 h) could reverse the Aβ-induced impairment of trafficking in DIV7 hippocampal neurons cultured under basal, unstimulated conditions. First of all, we tested the effects on pGOLT vesicles speed of mirtazapine alone and we found no difference with respect to control cultures treated with the DMSO vehicle (Supplementary Figure 2). When mirtazapine was co-applied with Aβ, we observed in proximal apical and basal dendrites no significant change in the percentage of static vesicles with respect to cultures treated with Aβ + DMSO (Figure 4A and Table 1). However, in high order dendrites, the percentage of static spots was significantly increased in cultures treated with Aβ + mirtazapine, with respect to control cultures (static spots: 42.6 ± 5.0% in DMSO vs. 58.5 ± 13.0% in Aβ + DMSO and 71.0 ± 6.0% in Aβ + mirtazapine; *p* = 0.004). Notably, the significant reduction in the anterograde spots observed in higher order dendrites after Aβ + DMSO treatment was not recovered by mirtazapine (29.0 ± 7.0% in DMSO; 5.6 ± 3.0% in Aβ + DMSO; 9.0 ± 3.0% in Aβ + mirtazapine; *p* = 0.02; Figure 4A and Table 1). A positive effect of mirtazapine included the regaining of the fastest spots with higher net distance covered in 10 min (Figure 4B) and the rescue of the population variance average velocity of pGOLT spots, which was contracted after Aβ treatment (Figure 4C). Of note, mirtazapine promoted a significant increase in pGOLT spot velocities only in proximal apical processes (0.09 ± 0.010 μm/min for Aβ + Mirta, vs. 0.05 ± 0.005 μm/min for DMSO; *p* = 0.0088 or 0.05 ± 0.007 μm/min for Aβ; *p* = 0.0182; Table 2). Moreover, mirtazapine induced a recovery to basal levels of the number of pGOLT spots in the three dendritic regions, which were strongly reduced by the Aβ + DMSO treatment (*p* < 0.05; Figure 4D). In conclusion, in unstimulated cultures mirtazapine induced a recovery of the number of pGOLT spots and regained the fastest pGOLT movements but the percentage of anterogradely moving pGOLT spots remained as low after Aβ treatment and there was a strong increase in static vesicles in higher order dendrites.

**FIGURE 5:**
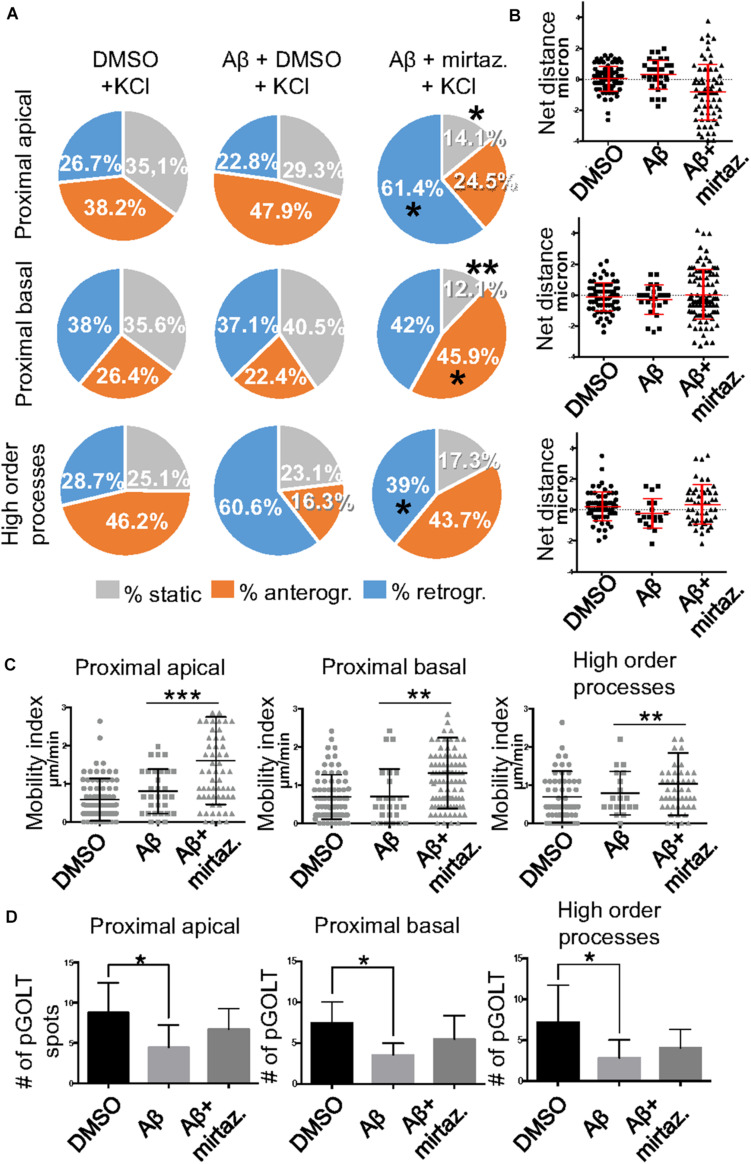
Effects of Aβ and mirtazapine on pGOLT vesicle trafficking in 50 mM KCl-stimulated cultures. **(A)** Pie charts representation of the average percentage of anterograde, retrograde, and static pGOLT spots after KCl stimulation (50 mM, 5 min). Data were obtained from 4 to 5 segments in proximal apical, proximal basal, and higher order processes. DMSO: *n* = 8; Aβ + DMSO: *n* = 7; Aβ + mirtazapine: *n* = 10. **(B)** Scatter plot representation of net distance of pGOLT spots in KCl conditions in proximal apical, proximal basal, and higher order processes after 4 h of treatments. **(C)** Mobility index calculated as the absolute net distance traveled by pGOLT spots in apical segments, basal segments, and higher order segments. DMSO: *n* = 8, *p* = 0.0004; Aβ + DMSO: *n* = 7, *p* = 0.0025; Aβ + mirtazapine: *n* = 10, *p* = 0.0173. **(D)** Average of the number of pGOLT spots in the different segments. DMSO: *n* = 8; Aβ + DMSO: *n* = 7; Aβ + mirtazapine: *n* = 10. Following D’Agostino-Pearson’s normality test, we performed a one-way ANOVA test or Kruskal Wallis test. *p ≤ 0.05; **p ≤ 0.01; ***p ≤ 0.001.

Since in unstimulated cultures mirtazapine was unable to rescue the impairment of the anterograde trafficking induced in distal dendrites by short-term Aβ treatment, in the subsequent series of experiments we investigated how neurons were affected by Aβ in the presence of sustained neuronal activity and if mirtazapine could have a beneficial effect under these conditions (Figure 5). In cultures stimulated with high K^+^ (50 mM, 5 min), Aβ + DMSO treatment did not significantly change the percentage of pGOLT spots with a static behavior or those moving anterogradely or retrogradely with respect to DMSO-treated cultures in proximal dendritic compartments (Figure 5A). However, similarly to unstimulated cultures, we observed in higher order dendrites, a trend at the limit of statistical significance toward a reduction in anterograde movements (from 46.2 ± 12.0% with DMSO + KCl to 16.3 ± 14.0% in Aβ + KCl, *p* = 0.0754; Figure 5A and Table 3) and an increase in retrograde spots following Aβ treatment (from 28.7 ± 9.0% with DMSO + KCl to 60.6 ± 16.0% in Aβ + KCl; Figure 5A). Interestingly, we found reduced variability of the net distance covered for both anterograde (positive values, Figure 5B) and retrograde pGOLT spots (negative values, Figure 5B). In particular, we observed that the number of fastest spots (i.e., those with longest net distance) was reduced in cultures treated with Aβ + KCl (Figure 5B), although the mobility index of pGOLT spots in cultures treated with Aβ + DMSO and KCl did not change with respect to controls (Figure 5C). Finally, we observed a significant decrease in the number of pGOLT spots following Aβ + DMSO + KCl treatment with respect to cultures incubated with DMSO + KCl (Figure 5D).

**TABLE 3:** Summary of the pGOLT dynamics in high KCl-stimulated cultures.

		**DMSO**	**Aβ + DMSO**	**Aβ + mirtazapine**
Proximal apical	Static	35.1 ± 6.6%	29.3 ± 6.7%	14.1 ± 7.0%*^1^
	Anterograde	38.2 ± 11.0%	47.9 ± 9.0%	24.5 ± 9.0%
	Retrograde	26.7 ± 6.0%	22.8 ± 9.0%	61.4 ± 11.0%*^2^
Proximal basal	Static	35.6 ± 6.0%	40.5 ± 13.0%	12.1 ± 4.0%*^3^
	Anterograde	26.4 ± 7.0%	22.4 ± 8.4%	45.9 ± 9.5%*^4^
	Retrograde	38 ± 10.0%	37.1 ± 10.0%	42 ± 8.0%
Higher order	Static	25.1 ± 6.0%	23.1 ± 13.0%	17.3 ± 8.0%
	Anterograde	46.2 ± 12.0%	16.3 ± 14.0%	43.7 ± 12.0%
	Retrograde	28.7 ± 9.0%	60.6 ± 16.0%	39 ± 12.0%*^5^

*Average percentage ± standard error of the mean (SEM) of static, anterograde, and retrograde vesicles for each neuron in control (DMSO) or after treatment with Aβ or Aβ + mirtazapine after treatment with high K + ^∗^1: *p* = 0.04, for Aβ + mirtazapine + KCl vs. Aβ + DMSO + KCl for static spots in proximal apical processes. ^∗^2: *p* = 0.03, for Aβ + mirtazapine + KCl vs. Aβ + DMSO + KCl for retrograde spots in proximal apical processes. ^∗^3: *p* = 0.01, for Aβ + mirtazapine + KCl vs. DMSO + KCl for static spots in proximal basal processes. ^∗^4: *p* < 0.05, for Aβ + mirtazapine + KCl vs. Aβ + DMSO + KCl for anterograde spots in proximal basal processes. ^∗^5: *p* < 0.05, for Aβ + mirtazapine + KCl vs. Aβ + DMSO + KCl for retrograde spots in higher order processes.*

**FIGURE 6:**
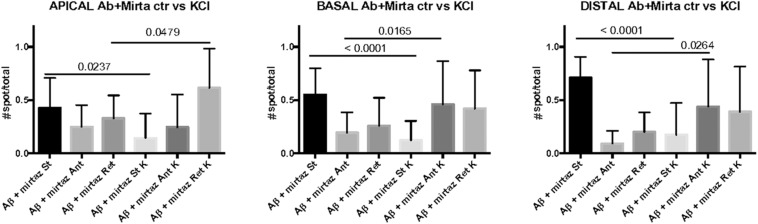
Comparison of number of pGOLT spots in control conditions and after high K^+^ treatment. The same hippocampal neurons were treated with Aβ + mirtazapine and recorded in time lapse before and after entering depolarizing conditions (K+, KCl, 50 mM, 10 min). The histograms represent the occurrence of static (St) or mobile anterograde (Ant) or retrograde (Ret) pGOLT vesicles in apical, basal, and in high order processes (distal), as indicated. Result of the statistical analysis between control and high K+ groups is indicated (*t* test). *N* = 7–18 neurons.

In cultures stimulated with high K^+^ and challenged with Aβ, mirtazapine had a general stimulating effect on the mobilization of pGOLT spots. In fact, in both proximal apical and basal processes, mirtazapine induced a significant reduction in the percentage of static spots (*p* < 0.04; Figure 5A). In particular, in proximal apical segments the percentage of static pGOLT spots was significantly reduced from 29.3 ± 6.7% in Aβ + DMSO + KCl to 14.1 ± 7.0% in Aβ + mirtazapine + KCl (*p* = 0.048), and, in the same conditions, the percentage of retrograde vesicles was significantly increased from 22.8 ± 9.0% in Aβ to 61.4 ± 11.0% in Aβ + mirtazapine (*p* = 0.03). In basal proximal dendrites there was a significant decrease in static pGOLT spots from 40.5 ± 13.0% in Aβ + DMSO + KCl to 12.1 ± 4.0% in Aβ + mirtazapine + KCl (*p* < 0.01) along with an increase in anterograde pGOLT spots from 22.4 ± 8.4.0% in Aβ + DMSO + KCl to 45.9 ± 9.5% in Aβ + mirtazapine + KCl (*p* < 0.05). In higher order processes, mirtazapine reverted the Aβ-induced decrease in retrograde mobility of pGOLT spots from 60.6 ± 16.0% in Aβ + DMSO + KCl to 39.0 ± 12.0% in Aβ + mirtazapine + KCl (*p* > 0.05; Table 3). In support of the idea that mirtazapine could have a stimulating effect on Golgi vesicles trafficking, we found that mirtazapine increased the number of pGOLT spots that moved for a significantly longer net distance in each of the three dendritic compartments considered (Figures 5B,C). In particular, in all dendritic segments analyzed, the pGOLT mobility index was significantly higher in Aβ neurons treated with mirtazapine with respect to neurons treated with Aβ only (*p* < 0.001 for proximal apical and *p* < 0.01 for proximal basal and high order processes; Figure 5C). Moreover, mirtazapine treatment induced a recovery of the quantity of pGOLT spots per segment, which was reduced after incubation with Aβ (Figure 5D). Finally, by time-lapse recording of pGOLT spots in the same Aβ + mirtazapine-treated hippocampal neurons before and after KCl depolarization, we found that depolarization induced a significant increase in the mobility of retrograde pGOLT spots in proximal apical processes (*p* = 0.0479) and in anterograde spots in basal and distal processes (*p* = 0.0165 and *p* = 0.0264, respectively) with a corresponding significant reduction in the number of static vesicles with respect to mirtazapine (Figure 6).

These results indicate that neuronal depolarization induced distinct responses in proximal apical, proximal basal, or higher order processes in conditions of impaired trafficking induced by Aβ treatment, suggesting regional distinct effects of mirtazapine overcame the Aβ-induced reduction in the number and mobility of pGOLT spots.

### Pharmacological Recovery of Aβ-Induced Neurite Atrophy

**FIGURE 7:**
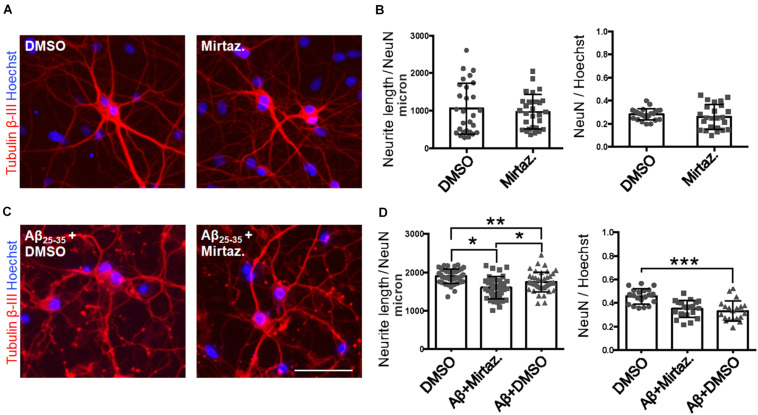
Rescue of Aβ-induced dendritic atrophy by mirtazapine treatment. **(A)** Representative microscopy images (10X) of DIV7 hippocampal neurons after 24-h treatment in DMSO **(left)** or mirtazapine (10 μM, 4 h **right**), stained with anti-Tubulin β-III (red) and Hoechst (blue). Scale bar = 50 μm. **(B)** Scatter plot of total neurite length for each neuron (**left**) expressed in μm/neuron, and neuronal density expressed as the ratio between the number of NeuN positive neurons on the total number of cells (**right**). Each dot represents an image in the different conditions, *n* = 24–25 fields per condition. **(C)** Representative images (10X) of DIV7 hippocampal neurons after 24 h of Aβ + DMSO **(left)** or Aβ + mirtazapine treatment **(right)**, stained with anti-Tubulin β-III and Hoechst. Scale bar = 50 μm. **(D)** Scatter plot of neurite length for each neuron **(left)** expressed in μm/neuron, and neuronal density expressed as the ratio between the number of neurons on the total number of cells **(right)**. Each dot represents an image in the different conditions, *n* = 40–44 fields per condition. Following D’Agostino-Pearson’s normality test, we performed an unpaired two-tailed *t*-test or one way ANOVA. *p ≤ 0.05; **p ≤ 0.01; ***p ≤ 0.001.

Considering the effects of Aβ and mirtazapine on vesicle trafficking, and the role of the Golgi apparatus in neuronal development and in the maintenance of dendritic arborization, we hypothesized that a pharmacological treatment with mirtazapine could reduce the Aβ-induced damage in cultured rat hippocampal neurons (Figure 7). Therefore, by applying the same methods used in Figure 2, we measured the total neuritic length (TNL) and the percentage of surviving neurons after 4-h treatment with Aβ_25__–__35_ alone (with DMSO), or in the presence of mirtazapine. Mirtazapine treatment alone (10 μM, 4 h) did not affect the TNL (1058 ± 128 for DMSO, vs. 971 ± 87 μm/neuron mirtazapine) nor the ratio of the number of neurons on total cell number with respect to cultures treated with the vehicle only (0.28 ± 0.01 NeuN/DAPI positive cells for DMSO, vs. 0.26 ± 0.02 for mirtazapine; Figures 7A,B). In a second set of experiments, Aβ + DMSO incubation (10 μM, 4 h) caused a significant blunting of TDL and reduction of surviving neurons with respect to DMSO controls (*p* = 0.0068 for TDL; *p* ≤ 0.001 for neuronal survival; Figures 7C,D). Co-application of Aβ and mirtazapine (both 10 μM, 4 h) to hippocampal neuronal cultures demonstrated a significant neuroprotective effect of mirtazapine with respect to Aβ-treated cultures on total neuritic length (1603 ± 47 μm/neuron in Aβ + DMSO, vs. 1754 ± 40 μm/neuron in Aβ + mirtazapine; *n* = 3 independent cultures; *p* = 0.0154; Figures 7C,D). The recovery effect was incomplete because cultures treated with mirtazapine showed TNL values significantly lower than control cultures treated with only DMSO (1754 ± 40 μm/neuron in Aβ + mirtazapine vs. 1906 ± 28 μm/neuron in DMSO; *n* = 3 independent cultures; *p* ≤ 0.05; Figures 7C,D). Moreover, mirtazapine treatment was not sufficient to protect against Aβ-induced cell death with respect to control (0.24 ± 0.02 for Aβ + DMSO vs. 0.25 ± 0.02 for Aβ with mirtazapine, not significantly different; Figure 7D right). In Figure 7, TDL data shown for the DMSO condition were different between Figures 7B,D which were carried out at different times. We previously reported that variations in the culture density can affect the TDL (Nerli et al., 2020). However, irrespective of the actual value, the validity of each experiment is given by the comparison to its own control.

## Discussion

**FIGURE 8:**
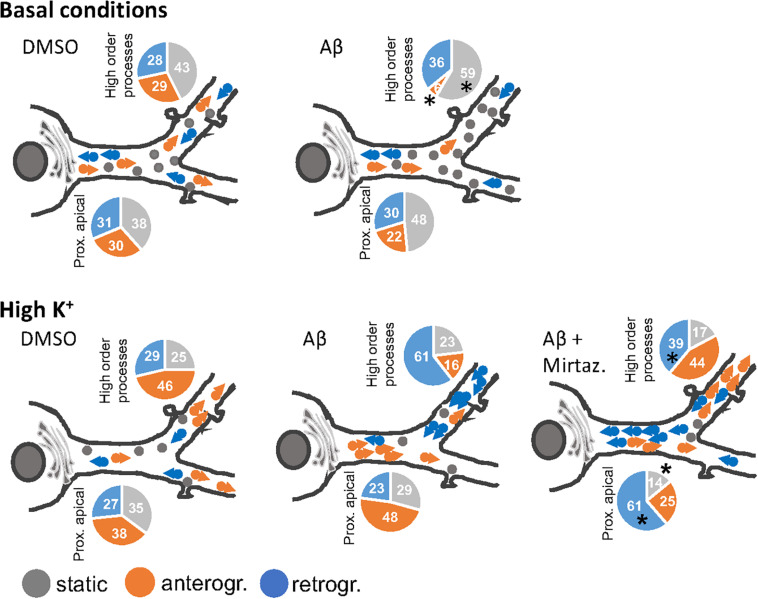
Model of the effects of a short-term challenge with Aβ oligomers on the trafficking of Golgi-like spots in the apical dendrites of rat hippocampal neurons and its rescue with mirtazapine treatment (the effects on basal dendrites are omitted for simplicity). The pie charts indicate percentage of static, anterograde, or retrograde moving pGOLT spots observed in living neurons, asterisks indicate significant changes induced by mirtazapine with respect to Aβ.

In this work we demonstrated that short-term (4 h) treatment with Aβ oligomers led to a decrease in anterograde trafficking of Golgi-like organelles in dendrites of hippocampal neurons. In neurons cultured under basal (unstimulated) conditions, the reduced percentage of Golgi vesicles undergoing anterograde trafficking is accompanied by an increase in the percentage of static Golgi spots and the effect is modest in proximal apical basal dendrites and much more marked in higher order dendrites. In contrast, in cultures stimulated with 50 mM KCl, the reduction in anterograde trafficking is visible only in higher order dendrites and is also accompanied by an increase in the percentage of static Golgi spots (summarized in Figure 8). We also showed the efficacy of mirtazapine in limiting the early damage induced by Aβ oligomers in hippocampal neurons, by increasing the total number and the general motility of Golgi spots in both an anterograde and retrograde direction, and reducing the percentage of static Golgi vesicles (Figure 8). In addition, mirtazapine largely reverted the neuronal atrophy induced by 24-h treatment with Aβ oligomers, suggesting that this drug is able to recover, at least in part, the neuronal damage by improving trafficking of the vesicles involved in the secretory route.

In this study, all experiments were carried out using the 11-amino acid sequence of Aβ25–35 which corresponds to the functional domain of the full-length Aβ1–40/42 peptides. The initial sequence of this short peptide fragment encodes a hydrophilic domain (Aβ25–28) involved in the formation of a β-sheet structure, while the terminal half is constituted by a hydrophobic domain (Aβ29–35) (Millucci et al., 2009). Similar to the Aβ1–42 or Aβ1–40 fragments, Aβ25–35 can form early intermediate aggregates such as monomeric or oligomeric soluble forms, or insoluble fibrils which are tightly associated with disease pathogenesis (Selkoe and Hardy, 2016). In particular, we chose to use Aβ25–35 because its presence was demonstrated in senile plaques of AD brains (Kubo et al., 2002), and it is the shortest fragment able to form large β-sheet fibrils, retaining the toxicity of the full length Aβ1–40/42 peptides (Kubo et al., 2002; Naldi et al., 2012).

### Effect of Aβ and Mirtazapine on Golgi Vesicle Trafficking

We found that even a short-term Aβ treatment of 4 h can cause a reduction in the number of Golgi vesicles in dendrites. The reduction in neurite outgrowth caused by long-term Aβ treatment might be associated with an overall reduced number of Golgi outposts (GOPs), as observed in other neurodegenerative disease models (Chung et al., 2017). Proper organization of both GOPs and Golgi satellites is fundamental for post-Golgi trafficking and dendrite elongation (Horton and Ehlers, 2003a; Horton et al., 2005).

In our work, we have studied the acute effect of Aβ and mirtazapine on the trafficking properties of pGOLT vesicles in hippocampal neurons. pGOLT-expressing neurons exhibited discrete spots in the soma and dendrites that were partially co-localized with antibodies targeting GM130 and TGN38, in line with previous findings (Mikhaylova et al., 2016). By employing a quantitative live imaging experiment analysis, we found that pGOLT vesicles were highly mobile in the perisomatic region, while they tended to become more static or oscillate in the dendrites. In particular, under basal conditions, pGOLT-transfected neurons were characterized by an equal fraction of static, retrograde, and anterograde pGOLT vesicles, distributed along the entire dendritic tree. Short incubation with Aβ oligomers had a strong effect on vesicle trafficking, supporting the known effects of Aβ on molecular motors (Vicario-Orri et al., 2015; Gan and Silverman, 2016; Gan et al., 2020). In fact, we observed a significant reduction in the number of pGOLT vesicles as well as significant impairment in their mobility in both directions with respect to the control in all compartments analyzed. After mirtazapine treatment, vesicles were more numerous, and had higher motility, covering significantly larger net distances with respect to control neurons.

We investigated the motility of pGOLT vesicles after depolarization with high K^+^, a treatment that is known to mobilize secretory vesicles and massively reinforce signaling following synaptic activation. In control conditions, membrane depolarization with high K^+^ strongly mobilized pGOLT anterograde trafficking. Similarly, in neurons treated with Aβ for only 4 h, high K^+^ was indeed sufficient to promote pGOLT vesicle mobilization, suggesting that in this early phase, the Aβ injury is still reversible. In fact, mirtazapine co-applied with Aβ was sufficient to positively impact the occurrence of pGOLT vesicles and significantly change their mobility, thus suggesting a positive effect of this drug on the molecular mechanisms that were impaired in Aβ-injured neurons. In addition, mirtazapine-treated neurons were visibly more trophic, with a larger membrane surface, and displayed a larger number of smaller pGOLT vesicles with respect to control. In the presence of KCl-induced neuronal depolarization, mirtazapine significantly sped up long-distance retrograde trafficking in apical and distal endings. After KCl, in Aβ, the percentage of static vesicles was similar to the vehicle, while in higher order processes the percentage of retrograde vesicles was strongly increased. In addition, mirtazapine induced decreased static pGOLT vesicles favoring retrograde transport toward the soma, with the exception of higher order processes where we observed a decrease in the percentage of retrograde vesicles. Moreover, mirtazapine induced a significant increase in pGOLT vesicles’ mobility index compared to Aβ-treated cultures.

The different dynamics observed in the dendrites of hippocampal neurons after KCl further confirmed the fact that primary dendrites exhibit a distinct trafficking mechanism compared to the higher order dendrites, as shown in basal condition (van Beuningen and Hoogenraad, 2016; Figure 8). Moreover, it is known that Aβ treatment may alter the ionic concentration within the neurons, interfering directly with the ion channels and pumps or intracellular organelles. In fact, several studies proved the capability of Aβ to form cationic channels permeable for Ca^2+^ in the membrane, leading to the disruption of Ca^2+^ homeostasis (Arispe et al., 1993) and further impairment of mitochondrial activity (Dong et al., 2016). The imbalance in Ca^2+^ may be further enhanced by the depolarizing effects of KCl, leading to strong activation of voltage-gated Ca^2+^ channels and synaptic activity. Ca^2+^ dyshomeostasis may interact with the activity of pGOLT, characterized by the transmembrane domain of the protein Calneuron-2, known as the Ca^2+^ sensor (Mikhaylova et al., 2016). It has been observed that sustained intracellular Ca^2+^ levels induced by high frequency stimulation by KCl prevents the inhibition activity of Calneuron-2 on the enzyme Phosphatidylinositol 4-OH kinase IIIβ (PI-4Kβ) leading to enzyme activation, increased Golgi-to-plasma membrane trafficking, and therefore retromer trafficking (Mikhaylova et al., 2009). The Calneuron-2 mechanisms may explain the fact that in Aβ-treated pGOLT-transfected neurons, high K^+^ led to a strong increase in retrograde vesicles in higher order processes, which is indicative of synaptic activity and endosome-to-TGN trafficking. On the other hand, mirtazapine co-application with Aβ recovered correct trafficking in distal segments, leaving an open question regarding which intracellular mechanisms, that re-establish the vehicle rates of retromer trafficking, are induced by this antidepressant.

### Differential Behavior of Vesicles in the Different Compartments of the Dendritic Arbor

It is not known whether Golgi transport mechanisms are equally regulated in the different regions of dendritic arborization and if they are functionally independent and with unique trafficking properties. Our data globally confirm the view that different segments of neuronal arborization are functionally independent and follow different trafficking rules. In particular, in resting cultures, Aβ affected the trafficking of pGOLT spots mainly in proximal apical and proximal basal processes, and both these effects were recovered by mirtazapine in the same regions (Figure 8). On the contrary, in higher order processes, Aβ significantly decreased the number of anterograde vesicles compared to control and mirtazapine increased the percentage of static vesicles, with little effect on their mobility (Figure 8). Interestingly, KCl-evoked depolarization had no effect on pGOLT spots in proximal segments, while the combination of KCl and Aβ treatments affected spots localized in apical segments and in distal endings, that are, however, differently mobilized, in anterograde and retrograde fashion, respectively. In conclusion, in proximal apical dendrites, mirtazapine partially restored vesicle mobility, presumably allowing the anterograde trafficking of vesicles necessary for the proper functionality of the neurons. Moreover, in higher order processes, mirtazapine seems to exert a dominant effect leading to an increase in Golgi outposts in distal dendrites, where elongation occurs. Regarding retrograde trafficking, mirtazapine was able to stimulate the spots mainly located in apical and distal endings, with no effect on spots on basal segments.

These data underline the differences between higher order dendrites and primary dendrites that originate from the soma and extend until the first branching point (Ye et al., 2007). Interestingly, it has already been described that organelles close to perinuclear regions may exhibit distinct functionality from those present in distal dendrites, since the concentrations of ions and proteins between these two compartments are different (Prydz et al., 2008; Britt et al., 2016).

### Effect of Aβ and Mirtazapine on Neurite Outgrowth

Mirtazapine is a noradrenergic and serotoninergic tetracyclic antidepressant, which in our experiments had no effect on neurite length or neuronal density of naïve hippocampal neurons, while it significantly counteracted the neuronal atrophy induced by Aβ, with no effect on neuronal loss. Aberrant neurite morphology caused by protein toxicity is a common feature of neurodegenerative diseases. Multiple mechanisms are involved in causing Aβ-induced neurodegeneration (Resende et al., 2007). Among others, Aβ oligomers affect the structure and function of molecular motors required for neurite elongation, trafficking, and sorting of vesicles essential for synaptic function (Brady and Morfini, 2017; Gan et al., 2020). In particular, Aβ induces Tau hyperphosphorylation and disengagement from microtubules affecting cargo transport, inducing deficits of neuronal protein transport, further leading to disruption of neuronal polarity (Ballatore et al., 2007). Furthermore, Aβ affects HDAC functioning, altering the acetylation of cytoskeleton proteins (Hubbert et al., 2002; Cohen et al., 2011) essential for microtubules dynamics.

The observed protective effects of mirtazapine on Aβ-induced neuronal atrophy can be explained by its multi-target way of action. In contrast to other antidepressants, mirtazapine does not inhibit the norepinephrine reuptake but rather antagonizes the α_2_-heteroreceptors in serotoninergic terminals. Additionally, mirtazapine acts as a blocker of 5-HT_2_ and 5-HT_3_ receptors, while promotes 5-HT_1__A_-mediated transmission (de Boer, 1996). In particular, 5-HT_1__A_ receptors were found mainly expressed in the soma and dendrites of CA1 pyramidal neurons (Ferreira et al., 2010). Serotonin production and expression of receptors, in particular 5-HT_1__A_ and 5-HT_7_, are involved in shaping hippocampal circuits, and the activation of 5-HT_1__A_ receptors improves neurite outgrowth of secondary neurites (Fricker et al., 2005; Rojas et al., 2014), suggesting a possible mechanism of action of mirtazapine. Moreover, the protective mechanism induced by mirtazapine may be mediated by promoting BDNF expression and release (Rogóz et al., 2005) as well as by promoting HDAC-related mechanisms (Ookubo et al., 2013).

### Use of Mirtazapine in Alzheimer’s Patients

Depression, often associated with severe weight loss, insomnia, and anxiety, is a comorbidity frequently found in patients affected by Alzheimer’s disease (Cassano et al., 2019). Unfortunately, as most antidepressant drugs were found to be ineffective, depression often presents as resistant to treatment (Elsworthy and Aldred, 2019). Among various possible explanations for the lack of efficacy of antidepressants in AD, a major hypothesis is that depression becomes resistant to treatment as a consequence of neurodegenerative events occurring at advanced stages of the pathology (Elsworthy and Aldred, 2019). To complicate this picture, meta-analysis studies have identified depression as a risk factor for AD, in that patients with a previous history of depression were more likely to develop AD later in life (Ownby et al., 2006; Tan et al., 2019). There is therefore an urgent need to understand how antidepressants may impact the cellular mechanisms underlying AD and its associated psychiatric symptoms.

Studies regarding the clinical use of mirtazapine treatment in AD are controversial. In three AD patients, mirtazapine was reported to promote a complete remission of depression, anhedonia, weight loss, sleep disturbances, and anxiety although memory deficits persisted (Raji and Brady, 2001). A 12-week open-label pilot study showed significant improvement in Cohen-Mansfield Agitation Inventory-Short form (CMAI-SF) scores and Clinical Global Impression-Severity scale (CGI-S) scores in 13 out of 16 patients (81.25%; Cakir and Kulaksizoglu, 2008). However, a large double-blind, placebo-controlled clinical study for the treatment of depression in AD conducted on 326 subjects (111 controls, 107 mirtazapine, 108 sertraline) across 9 centers in the United Kingdom found no benefit of mirtazapine or sertraline compared to placebo at 13 and 39 weeks (Banerjee et al., 2011). On the other hand, full resolution of associated symptoms such as weight loss, sleep problems, and anxiety has been consistently reported in several studies (Raji and Brady, 2001; Urrestarazu and Iriarte, 2016; Franx et al., 2017). Interestingly, recent studies have investigated non-canonical effects of antidepressants on neurobiological mechanisms, demonstrating that various antidepressants, including mirtazapine (Sun et al., 2007) are able to downregulate amyloid-β peptide levels in the serum and brain of AD patients and transgenic animal models (reviewed in Cassano et al., 2019). Our results on the effects of mirtazapine Golgi trafficking add to this emerging trend of investigations in AD.

### Conclusion

In AD, transport deficits underlie neuronal dysfunction and synaptic loss. We propose that mirtazapine can exert protective effects against Aβ injury by acting on dendritic trafficking mechanisms that are required for the proper functioning of the anterograde secretory pathway as well as retrograde retromer trafficking in dendrites. Previous studies have shown that Aβ oligomers and neuroinflammation associated with AD impair axonal and dendritic retromer trafficking of BDNF, causing a downregulation in neurotrophin signaling, essential for neuronal development and maintenance of dendritic complexity (Poon et al., 2011; Gan and Silverman, 2016; Seifert et al., 2016; Carlos et al., 2017; Plá et al., 2017). In hAPP transgenic mice, the impairment in axonal retrograde trafficking induced an aberrant retention of endosomes in distal neurites and impaired endosome-TGN and lysosomal functioning (Tammineni et al., 2017). Concerning anterograde Golgi trafficking along the secretory pathway, a different outcome on BDNF or glutamate release in Aβ-treated neurons was described: while BDNF secretion is lowered by Aβ treatment, glutamate release remains unchanged, indicating a specific impairment of the protein secretory pathway (Plá et al., 2017). Thus, the efficacy of mirtazapine in restoring Golgi trafficking is promising for possible future employment in AD treatment.

## Data Availability Statement

The raw data supporting the conclusions of this article will be made available by the authors, without undue reservation.

## Ethics Statement

The animal study was reviewed and approved by the Local Ethical Committee of the University of Trieste on November 10th 2017 and was communicated to the Italian Ministry of Health, in compliance with the Italian law D. Lgs.116/92 and the L. 96/2013, art. 13.

## Author Contributions

ET and EF designed the study, wrote the first draft of the manuscript, and edited the final manuscript. GA and EF carried out the experiments and analyzed the results. All authors contributed to the article and approved the submitted version.

## Conflict of Interest

The authors declare that the research was conducted in the absence of any commercial or financial relationships that could be construed as a potential conflict of interest.

## Supplementary Material

Supplementary Figure 1 MTT assay dose-curve response of drug and vehicle concentrations on cell survival.

Supplementary Figure 2 Mobility of pGOLT vesicles after DMSO or mirtazapine treatment. **(A–C)** Examples of time-lapse images of pGOLT vesicles in cultures incubated with vehicle (DMSO) or treated with mirtazapine (MIRTAZ.), under basal conditions or in high KCl medium (post K+). **(D)** Quantification of mobility index in the proximal apical, proximal basal dendrites, or high order processes.

Supplementary Table 1 Antibodies used in this study.
