# Supplementary figures and images for: Amyloid-β Impairs Dendritic Trafficking of Golgi-Like Organelles in the Early Phase Preceding Neurite Atrophy: Rescue by Mirtazapine
Source: Front Mol Neurosci. 2021 Jun 3;14:661728. doi: 10.3389/fnmol.2021.661728 (PMC8209480; doi:10.3389/fnmol.2021.661728)

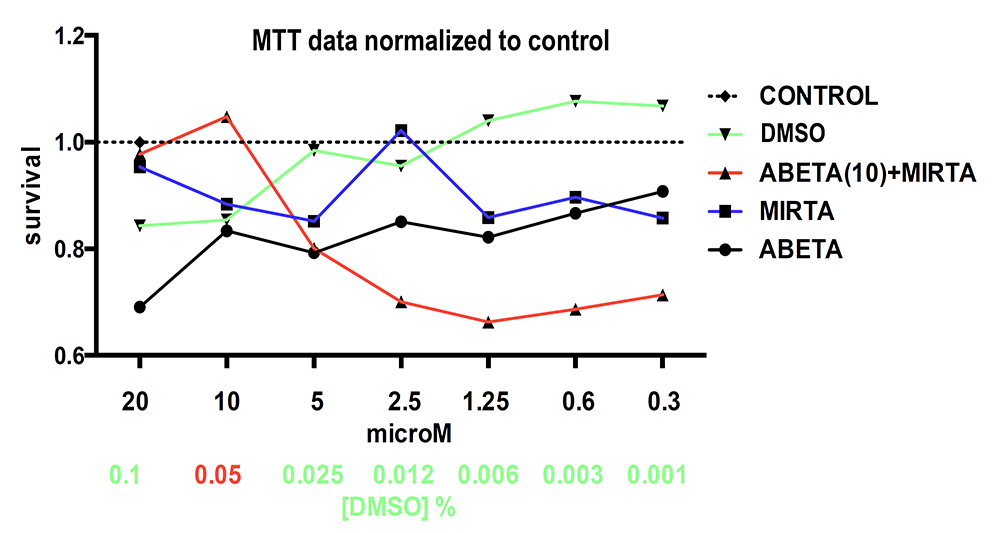

Supplement: Supplementary Figure 1 — MTT assay dose-curve response of drug and vehicle concentrations on cell survival. [file Image_1.TIF]

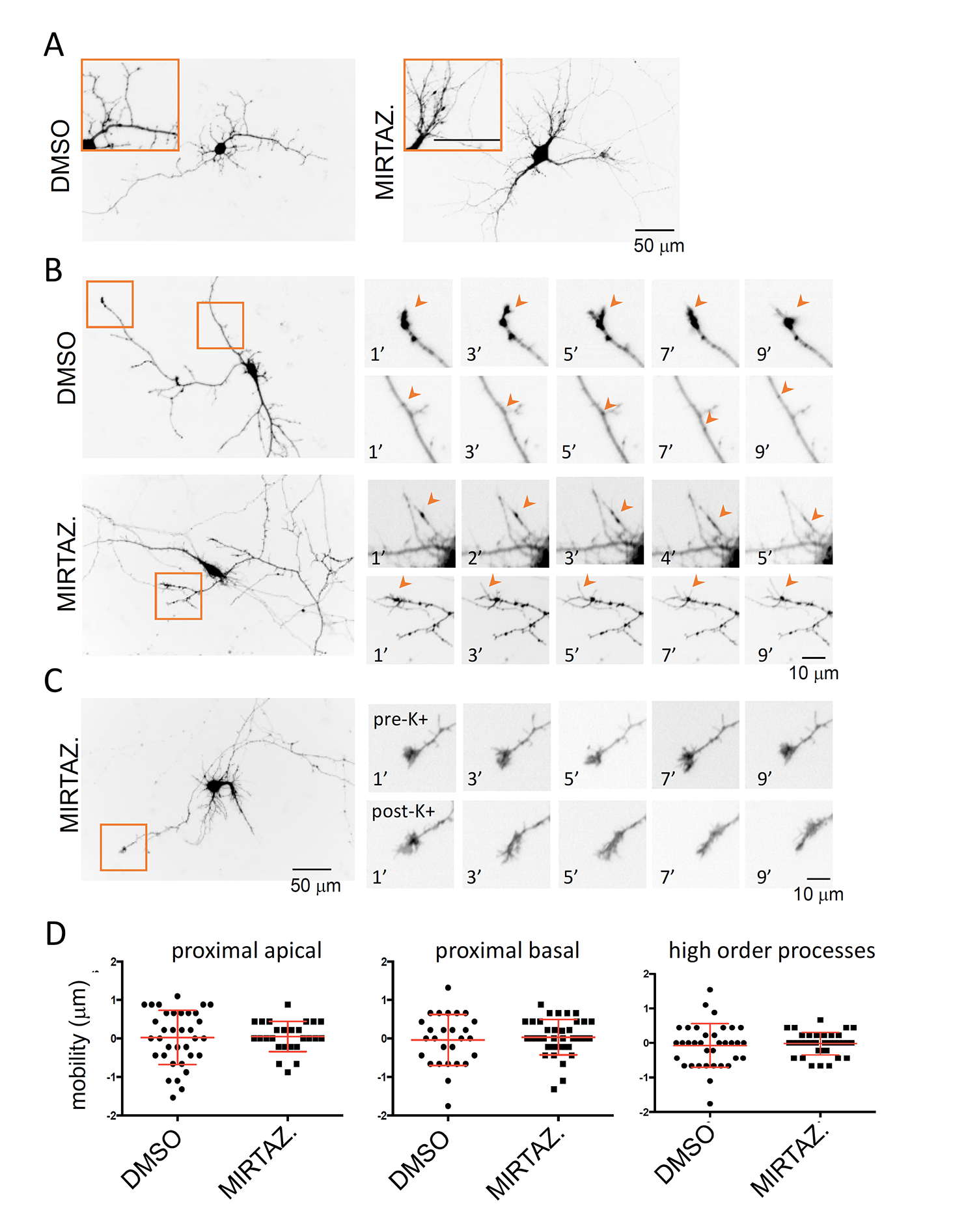

Supplement: Supplementary Figure 2 — Mobility of pGOLT vesicles after DMSO or mirtazapine treatment. (A–C) Examples of time-lapse images of pGOLT vesicles in cultures incubated with vehicle (DMSO) or treated with mirtazapine (MIRTAZ.), under basal conditions or in high KCl medium (post K+). (D) Quantification of mobility index in the proximal apical, proximal basal dendrites, or high order processes. [file Image_2.TIF]
