# Supplementary material for: Amyloid-β Impairs Dendritic Trafficking of Golgi-Like Organelles in the Early Phase Preceding Neurite Atrophy: Rescue by Mirtazapine
Source: Front Mol Neurosci. 2021 Jun 3;14:661728. doi: 10.3389/fnmol.2021.661728 (PMC8209480; doi:10.3389/fnmol.2021.661728)
Supplement: Supplementary Table 1 — Antibodies used in this study. [file Table_1.DOCX]

**Supplementary Table I. Antibodies used in this study.**

| **Antibody** | **Species/Type** | **Dilution** | **Company** |
| --- | --- | --- | --- |
| anti-βTubulin III | Rabbit pAb | 1:1000 | Sigma (Milan, Italy) |
| anti-NeuN | Mouse mAb | 1:1000 | Millipore (Burlington, MA, USA) |
| anti-GM130 | Mouse mAb | 1:250 | BD Transduction lab (San Jose, CA) |
| anti-TGN38/46 | Mouse mAb | 1:250 | Abcam (Cambridge, UK) |
| anti-LMAN1 | Chicken pAb | 1:250 | Sigma (Milan, Italy) |
| Anti-rabbit IgG AlexaFluor 568 | Goat pAb | 1:500 | Thermo Scientific Italia (Rodano (Milan), Italy) |
| Anti-rabbit IgG AlexaFluor 488 | Goat pAb | 1:500 | Thermo Scientific Italia (Rodano (Milan), Italy) |
| Anti-mouse IgG AlexaFluor 568 | Goat pAb | 1:500 | Thermo Scientific Italia (Rodano (Milan), Italy) |
| Anti-rabbit IgG AlexaFluor 488 | Goat pAb | 1:500 | Thermo Scientific Italia (Rodano (Milan), Italy) |
| Anti-chicken IgG AlexaFluor 488 | Rabbit pAb | 1:500 | Thermo Scientific Italia (Rodano (Milan), Italy) |
